# Nursing challenges for universal health coverage: a systematic review^1^

**DOI:** 10.1590/1518-8345.0933.2676

**Published:** 2016-04-29

**Authors:** Mariana Cabral Schveitzer, Elma Lourdes Campos Pavone Zoboli, Margarida Maria da Silva Vieira

**Affiliations:** 2 Post-doctoral fellow, Escola de Enfermagem, Universidade de São Paulo, São Paulo, SP, Brazil. Scholarship holder from Coordenação de Aperfeiçoamento de Pessoal de Nível Superior (CAPES), Brazil; 3 PhD, Associate Professor, Escola de Enfermagem, Universidade de São Paulo, São Paulo, SP, Brazil; 4 PhD, Associate Professor, Instituto de Ciências da Saúde, Universidade Católica Portuguesa, Porto, Portugal

**Keywords:** Nursing, Universal Access to Health Care Services, Humanization of Assistance, Primary Health Care, Review

## Abstract

**Objectives:**

to identify nursing challenges for universal health coverage, based on the findings of a systematic review focused on the health workforce' understanding of the role of humanization practices in Primary Health Care.

**Method:**

systematic review and meta-synthesis, from the following information sources: PubMed, CINAHL, Scielo, Web of Science, PsycInfo, SCOPUS, DEDALUS and Proquest, using the keyword Primary Health Care associated, separately, with the following keywords: humanization of assistance, holistic care/health, patient centred care, user embracement, personal autonomy, holism, attitude of health personnel.

**Results:**

thirty studies between 1999-2011. Primary Health Care work processes are complex and present difficulties for conducting integrative care, especially for nursing, but humanizing practices have showed an important role towards the development of positive work environments, quality of care and people-centered care by promoting access and universal health coverage.

**Conclusions:**

nursing challenges for universal health coverage are related to education and training, to better working conditions and clear definition of nursing role in primary health care. It is necessary to overcome difficulties such as fragmented concepts of health and care and invest in multidisciplinary teamwork, community empowerment, professional-patient bond, user embracement, soft technologies, to promote quality of life, holistic care and universal health coverage.

## Introduction

The route to Universal Health Coverage (UHC) and the Post-2015 development agenda is through the health worker^(^ 1 ^)^. This agenda includes reducing maternal mortality, end preventable deaths of newborns and under five-year-old children, end the epidemics of AIDS, tuberculosis, malaria and Neglected Tropical Diseases (NTDs), and ensure universal access to health care services^(^ 2 ^)^. All these objectives can be achieved using nurses and midwives strategically placed at the person-centered community-based level. 

Nurses and midwives are the largest category of health workforce, related to eighty percent of health services, and are also the frontline health workers. Nursing and midwives using a career pathway model of skill mix could be utilized to deliver health care and improve outcomes. However, affordable approaches to boost the performance of health workers are urgently required. The path towards UHC implies addressing the gaps in competency, quality, motivation, productivity and performance of health workforce^(^ 3 ^)^. 

Key practical steps proposed by WHO for Nursing and Midwifery are: increase interdisciplinary, multi-professional, nurse-led teams and leadership skills; empower nursing and midwifery workforce by clear role clarification, valid job description and professional recognition; implement positive work environments to improve motivation and retention^(^ 4 ^)^. 

One way towards these steps was the inclusion of the National Humanization of Health Care and Health Care Management Policy, also known as the National Humanization Policy (NHP) and/or HumanizaSUS, in different nurses and midwives' workplaces^(^ 5 ^)^. This unique policy is guided by values such as autonomy and empowerment of health users, responsibility among patients and health professionals, establishment of solidarity, the construction of cooperation networks and collective participation in the management process. The NHP operates the following devices: user embracement, unique therapeutic project or patient centred care, public health projects; qualified listening of health users and workers; among others.

Primary Health Care (PHC) is the gateway to the health system and organizes the network of services. Humanization permeates the work processes and the stakeholders of primary care^(^ 6 ^)^. Considering this relation, the objective of this study was to identify nursing challenges for UHC, based on the findings of a systematic review focused on the health workforce's understanding of the role of humanization practices in PHC.

## Method

The purpose of a systematic review is to enable the translation of the best scientific evidence into policies, practices and decisions in the healthcare context^(^ 7 ^)^. A mixed research synthesis by integrated design was used in this study. This design allows grouping the findings of both types of primary studies into thematic categories to reach meta-synthesis^(^ 8 ^)^. This integrated design uses PICo to guide data collection, a specific guide to extract information and to classify the quality of findings. 

In November 2013, the search was conducted using the following databases: PubMed, CINAHL, Scielo, Web of Science, PsycInfo, SCOPUS, DEDALUS and Proquest. The references of the articles selected for this review served as a source of new inclusions in the review process so-called reference of the reference^(^ 9 ^)^.

PICo was used to guide the systematic review question, these mnemonic identifies the key aspects Population, Phenomenon of Interest and Context^(^ 10 ^)^. In this review, Population was PHC Professionals, Phenomenon of Interest was Experiences of humanization practices and Context was PHC Settings. Adjusting the objectives of the study to PICo, the guiding research question of this systematic review was "What is the understanding of healthcare professionals with regard to the role of humanization practices in PHC?"

Brazilian NHP^(^ 5 ^)^ concepts of humanization are centered on core principles such as access, empathy, humanization of assistance, personal autonomy, holistic health and user embracement. Data collection used controlled search terms based on these concepts. The following keywords were used: Primary Health Care with others, separately: humanization of assistance, holistic care/health, patient centred care, user embracement, personal autonomy, holism, and health personnel attitude. 

Research articles were found in English and Portuguese from 1999 to 2011. The inclusion criteria for the study were: articles related to attitudes/beliefs of primary health care professionals regarding the use/practice of humanization practices. Articles were excluded if were about patients or students and/or studies done in hospital settings. The articles were organized using EndNote, which is an electronic reference manager. Each article received an identification number. The articles referring to the same study were treated as one and given a single identification number.

Two independent reviewers assessed the articles and the final selection was made by consensus, based on a comparison of the evaluation of both reviewers. The analysis results were organized using a modified version of the Data Extraction Guide for Quantitative and Qualitative Studies^(^ 11 ^)^. Knafl & Sandelowski^(11)^ tool provides guidance on how to transform raw data into data that can be systematically combined and analyzed. 

Information was extracted from each report in the following domains: research purposes and questions, theoretical framework, method and design, sampling strategy, sample composition, data collection and analysis techniques, techniques to optimize validity or minimize bias, techniques to protect human subjects, findings, and discussion^(^ 12 ^)^. Based on these domains each study was analyzed and classified as Strong, Good, Weak and Noisy (when there was lack of information).

As recommended for systematic reviews aimed at providing a metasynthesis by integrating the results of qualitative and quantitative studies (Mixed research synthesis)^(^ 9 ^,^ 13 ^)^, the quantitative findings were qualified. Findings were converted into a qualitative format in order to combine them, by themes. The data was organized into empirical categories that emerged from the analysis. Two independent reviewers assessed the categories in terms of their respective scope and definition, with disagreements also being resolved by consensus. 

The analysis of findings from the systematic review considered the quality and results of the studies. The Consolidated Criteria for Reporting Qualitative Research (COREQ) checklist was applied to improve the quality of the recommendations presented in this review^(^ 14 ^)^. 

## Results

**Figure 1:**
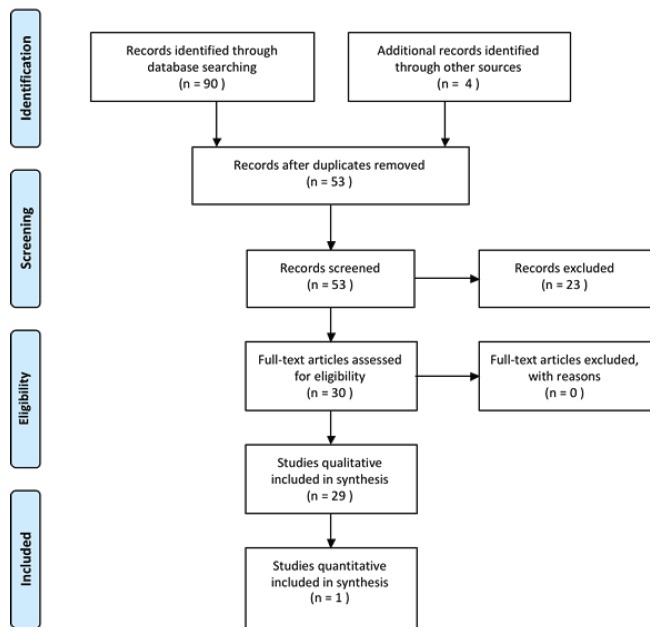
- Diagram of the process of inclusion and exclusion for all the studies in the systematic review^(15)^

From potential 90 references, reduced to 53 non duplicated studies, 30 studies were included and systematically reviewed: 29 qualitative and 1 quantitative as Figure 1 shows. Studies retrieved were undertaken between 1999 and 2011. One study was published in English and 29 in Portuguese. 

**Figure 2:**
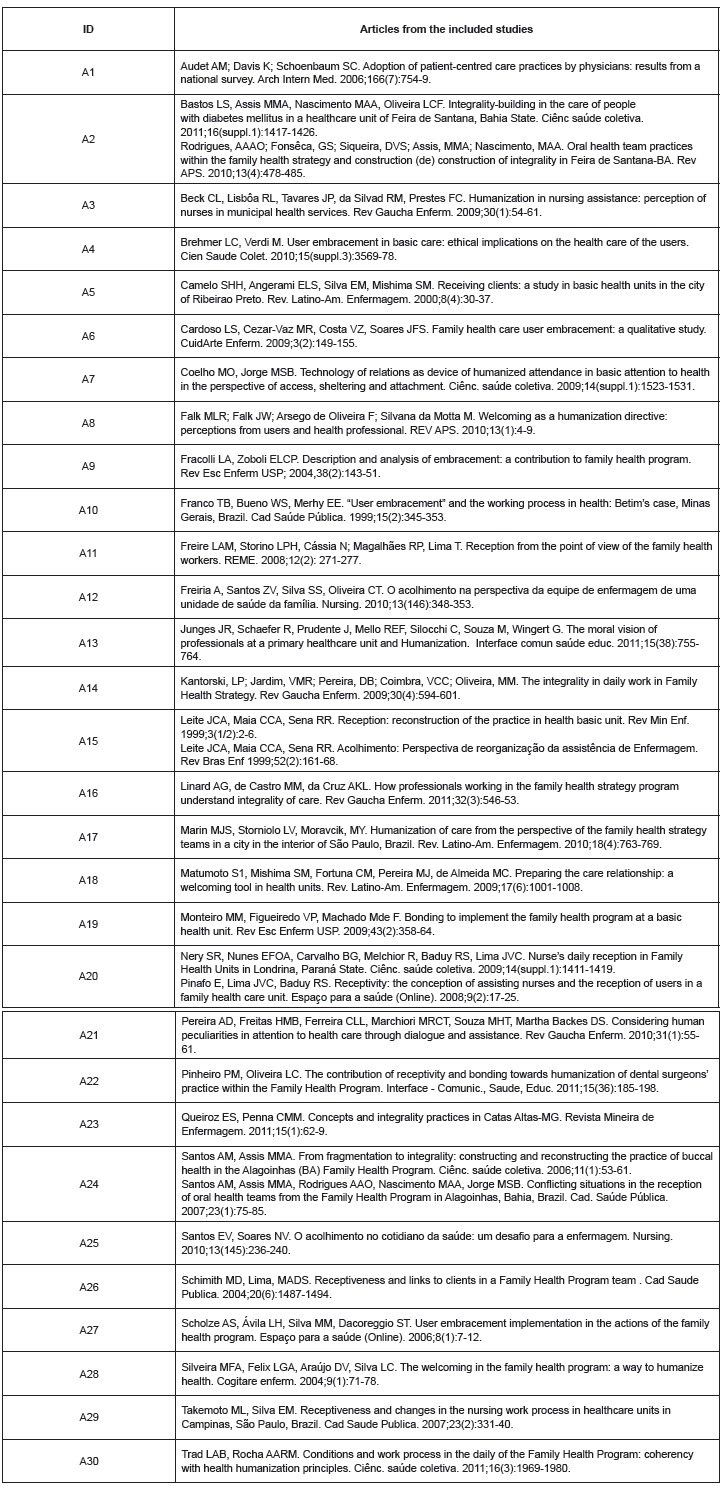
- Studies included in the systematic review on humanization practices

In total, studies included 1,179 PHC professionals from United States of America (A1) and Brazil (A2-30) as shown in Figure 2. Nearly 50% of these professionals were physicians from USA; the other 50% included professionals from Brazil, mostly nurses, auxiliary nurses, nursing technicians, physicians, community health agents, dentists and dentist auxiliaries and some administrative personnel.

Each study was analyzed by its quality and classified: 18 as Strong (A3, A5, A6, A8, A10, A11, A14, A17, A19, A20, A22, A24, A25, A26, A27, A28, A29, A30), 5 as Good (A2, A15, A16, A21, A23), 2 as Weak (A1, A12) and 5 as Noisy (A4, A7, A9, A13, A18).

The findings were aggregated in eight empirical categories: attitudes and beliefs; health care conceptions; barriers; facilitators; education and training; professional-patient bond; teamwork and provision of humanizing practices. In this article we describe the categories: health care conceptions; barriers; facilitators; education and training about humanizing practices and provision. In another paper we describe the other three categories that were identified in the review^(^ 16 ^)^. 

## Category 1: Conceptions of health and care in the humanization practices

This category included perceptions of health care professionals about conceptions of health and care related to humanization practices. This category was present in 17/30 studies.

Regarding the concept of health, more than 130 professionals reported the need to consider the patient in a physical, psychological and spiritual perspective and their social and cultural context, beyond the biological dimension (A3, A4, A7, A9, A16, A17, A20, A21, A23).

Regarding the concept of care, more than 60 workers mentioned the importance of individualization of care, recovery of light technologies, shared responsibility, user embracement, need to meet social problems and promote behavior changes (A14, A20, A22, A24, A28, A29).

Professionals mentioned and it was observed that the escape of the traditional curative model occurs when professionals seek to put in place a comprehensive conception of health, through the recovery of the essence of caring, accountability, sensitivity to listening, integrative perspective, use of light technologies, rescue of professional-patient bond, health practice that promotes behavior change and improves quality of life (A14, A20, A22, A24, A28, A29).

Professionals reported difficulties in meeting concepts of health and care related to uniqueness, autonomy and co-responsibility due to a health activity marked by individualism, complaint-conduct and fractionation of therapeutic acts (A2, A4, A7, A9, A13, A20, A21, A24, A29).

The search for productivity, lack of working conditions, inadequate conceptions of the health-disease, unjust social conditions of the community and workers' predetermined conceptions about the health users were cited as barriers to the achievement of universal care (A2, A7, A9, A26, A28).

## Category 2: Barriers of humanization practices

This category included perceptions of health care professionals about difficulties, barriers and limitations related to humanizing practices. This category was present in 23/30 studies. 

More than 700 workers reported factors related to: 1) Lack of feedback (A1); 2) Lack of reference and counter-reference (A1, A4, A2, A14, A15, A17, A30); 3) Excess of demand, inadequate physical space, lack of materials and professionals (A2, A3, A4, A11, A13, A15, A17, A14, A18, A20, A22, A24, A25, A26, A27, A28, A29 , A30); 4) Lack of support, partnership and attention from the County Health Department and community participation in meetings promoted by healthcare units (A3, A24, A26, A30); 5) Excessive bureaucratic work and lack of time (A3, A17, A20); 6) Inflexible working hours (A3, A14, A15, A18, A24); 7) Vertical Management (A4, A13, A20); 8) Dissatisfaction of professionals with working conditions (A2, A4, A20, A26, A28, A30); 9) Lack of educational groups, mental health professionals and other areas for referral (A9, A20); 10) Inadequate attitude of professionals and users about user embracement and humanization practices (A9, A10, A11, A13, A15, A17, A20, A22, A23, A24, A26, A27); 11) Lack of teamwork and definition of professional roles (A10, A13, A15, A20, A26); 12) Lack of user embracement and Family Health Program teams (A14, A19); 13) Problems related to unsatisfactory community social conditions (A9, A28, A29); 14) Lack of knowledge and specific training about Unified Health System and Family Health Program to work in Primary Health Care (A25, A30).

## Category 3: Facilitators of humanizing practices

This category included perceptions of health care professionals about facilitators and promoting factors related to humanizing practices. This category was present in 18/30 studies.

More than 300 workers mentioned factors related to: 1) Commitment of teams and responsibility with the community (A3, A14, A19, A30); 2) Participative management, including Unified Health System network, Family Health Program coordinators, Local Health Council, professional associations and third sector (A3, A10, A17, A24, A30); 3) User embracement practice over 5 minutes (A5); 4) Permanent discussions between the staff of the Health Unit to evaluate and reprocess user embracement (A10, A27); 5) Favorable working conditions such as more time, fewer people to attend, better physical space, more resources (A9, A12, A17, A18, A25, A30); 6) Community commitment and positive recognition verbalized by patients (A11, A20, A28); 7) Dialogue, professional-patient bond and better knowledge of community needs by a work process called ascription of the community (A14, A19, A20); 8) Understanding and acceptance of user embracement process by professionals and health users, patient availability of "giving vacancy to another patient more serious" (A15); 9) Presence of teamwork, performance evaluation and adequate division of activities between professionals (A10, A14, A15, A17, A19, A20, A27); 10) Continue education and training of health workforce professionals (A10, A17, A19, A25); 11) Use of protocols and record data of user embracement (A9, A10); 12) Flexibility for scheduling consultation times and less bureaucratic practice (A18); 13) Organization of programmatic groups, support groups and provision of other professional services (A3, A10); 14) Realization of public contracts to contribute to retention of professionals in enrolled areas, preventing turnover of personnel (A19).

## Category 4: Influence of education and training on humanizing practices

This category included perceptions of health care professionals regarding the influence of training in the context of humanizing practices, including considerations regarding graduation courses and continuing education. This category was present in 7/30 studies.

More than 60 professionals, including managers, reported a need for more training and greater awareness of humanizing practices (A15, A17, A25). From these, nine nurses highlighted the need for training related to conformation and operation of multidisciplinary teams to deploy user embracement (A15).

Studies have reported difficulties with training human resources to work in Primary Health Care and with humanizing practices (A2, A15, A17, A25, A30). Professionals from six healthcare units reported non-operation of the orientation training or that only some professionals can participate, highlighting the lack of training for dentists and nursing assistants (A30).

Professionals reported experiences of training/workshops about user embracement at work and humanization of care (A22, A30); nurse auxiliaries and community health agents mentioned frequent participation in health training and activities (A30). A Family Health Program team has Permanent Education project to train and qualify professionals in accordance with the principles of humanization policy (A17).

## Category 5: Provision of humanizing practices

This category included perceptions of health care professionals about provision of humanizing practices, how this is organized, who practices them and their consequences. This category was present in 26/30 studies. 

Professionals reported the implementation of humanizing practices in PHC, such as user embracement, integrality, access, bond, universality, patient-centered practice and extended clinic (A1, A5, A6, A9, A10, A11, A14, A15, A23, A24, A27, A29, A30).

Regarding user embracement, from its implementation and consequent reorganization of the work process, in a health care unit, changes were observed in accessibility to services, demonstrated through the extraordinary increase of general care delivery by the Unit over one year. Data on the unit show that efficiency increased by 600% [production/hours worked], related to the extraordinary increase in the yield by nurses, social workers and nursing assistants with the deployment of user embracement and the work reorganization process (A10).

In ten health care units it was observed that the actions related to the process of user embracement were to identify the patient's problem and propose an answer; refer patients to other services such as first aid, medical consultation, etc.; perform anamnesis towards the complaint; perform screening for immediate or mediate referral, according to pre-established number of consultations and severity of the complaint; perform physical examination and check vital signs, focus on the complaint; supervise the nursing assistant when it performs user embracement; oversee the station entrance door; distribute tickets to attend; perform medical or nursing consultation; change drug prescriptions; realize health guidelines orientation; take care of wounds; administer medications; perform qualified listening to meet patient needs; provide support to people seeking the service (A9).

The offer of humanizing practices changed the organization and division of labor between the teams of PHC, especially due to the implementation of user embracement (A5, A9, A13, A18, A26, A27, A30).

User embracement resulted in increased workload and teams performance with reorganization of the work process (A10, A22, A29). In some healthcare units user embracement was offered by all staff (A6, A10), in others it was in charge of the nursing team (A9, A15, A29) and in some units doctors were in the rear of user embracement (A10, A11, A15). User embracement brought changes in the work process of the nursing team, re-meaning care for nursing assistants (A10, A29).

In some consultations and in some units was observed that user embracement was practiced by the nursing assistant, carefully, showing availability, interest, involvement and response to health user needs (A5). Seventeen nurses reported that all employees dynamicalle and continuously performed user embracement at any time and place (A6). 

In ten health care units it was observed that, with respect to the professionals who deploy user embracement, in general, this activity is the responsibility of nurses and nursing assistants, with the rear of the medical professional; in some units, the community health worker was responsible for user embracement, It was observed that, each day, an average of forty user embracement practices took place, which lasted from three to fifteen minutes each (A9). Among fifteen workers, most confirmed doing user embracement, only two doctors said not to do it (A11). In nine health care units, there is great diversity in the composition of the work teams that have deployed user embracement: nursing assistants were at the frontline of this practice with the participation of nurses as technical reference; physicians act as support for unsolved cases, limited to 12 vacancies for consultation per physician, without bonding and accountability for this practice (A15).

Primary healthcare teams had doubts in implementing humanizing practices, especially in the organization of services and work teams related to user embracement (A9, A12, A20, A21, A22, A24, A26); being common to this practice to end in medical consultations, without changing staff practices or the management of the unit (A9, A26, A27, A29).

Workers and managers reported and it was also observed in some healthcare units that they did not offer humanizing practices, resulting in restricted and limited patient access, work focused on consultations and medications, being exclusive to medical specialties of medium and high complexity, low resolution, lack of light technologies, user embracement primarily being applied as a technique of reception, screening and referral, synonymous of emergency or waiting room; work processes focused on procedures and techniques, as well as work process divided by each specialty; attachment to the procedures and protocols; bureaucratic practice (A2, A4, A5, A7, A13, A15, A18, A28, A24, A26, A28, A29, A30).

## Discussion

Professionals' conception of health in relation to humanizing practices included the physical, social, psychological and spiritual perspectives in search of wellbeing and quality of life through an integrative care. The conception of care included the use of soft technologies, individualization of care, patient empowerment, co-responsibility, access to services, user embracement, reference and counter-reference, teamwork, adequate professional behavior, changes in work process, the demand to attend social problems and to create life changes opportunities to patients.

Since the late twentieth century, in the Brazilian context, different proposals, such as integrative care, health promotion, humanization practices, have sought to overcome technical, political and ethical impasses in health care^(^ 17 ^)^. As most of the studies included in this review were of Brazilian practices, it was not surprising to find concepts of health and care that include these proposals and practices.

Health promotion is based on an expanded concept of health and disease and its determinants. In this conception, health professionals and population are seen as subjects of the process and the focus of care is related to the way of life and working conditions of individuals and social groups and their impact on the health-disease process^(^ 18 ^)^.

Nursing theories also present expanded concepts of health and care, related to the bio-psycho-social-spiritual context, interpersonal relationships, holistic care, empowerment and health care needs. Just to present some examples: Florence Nightingale showed the relationship of the person and the environment to health, Martha Rogers continued to demonstrate this relationship and included the concept of holistic care, Rosemarie Parse increased the perception of a person including the spiritual domain, Madeleine Leininger included cultural aspects, Virginia Henderson the basic needs and self-care, Hildegard Peplau the importance of interpersonal relationships, Afaf Meleis the concept of transitions, Jean Watson the concept of a critical event and humane care and Wanda Horta the concept of basic human needs^(^ 19 ^-^ 21 ^)^.

Therefore, in the health promotion approach, nursing theories and professionals in PHC present neither idealized nor fragmented conceptions of health and care in relation to humanizing practices. Thus, nurses are encouraged to recognize and reaffirm the role of nursing related to promoting quality of life for those who no longer have the ability to care for themselves alone.

However, professionals also cited barriers to work according to these conceptions of health and care in order to deliver humanization practices. The barriers were not all present in a single practice, but were common at many units. It is a real challenge for teams, managers and health users to modify these barriers towards integral and universal care.

One of the biggest current ethical challenges in the Unified Health System is to produce therapeutic linkages to ensure health users, family and community the possibility of health care in adequate time^(^ 22 ^)^. Difficulties with access and referral put UHC in danger and also reflect on the health professional bond with health users and communities, especially in relation to confidence in PHC services.

On the other hand, professionals cited many facilitators of humanizing practices. The facilitators were more common in units that had performed user embracement longer, those which increased health user access to the unit and where meetings were held with all professionals to evaluate health care services.

The complexity of the problems and the organization of services in PHC require changes in professionals and health users' attitudes and values^(^ 23 ^)^. These changes also include providing adequate working conditions to enable the commitment of the teams and responsibility towards the community. Workers need specific training, and participatory management is needed, including health services, Local Health Council, professional organizations and the third sector, to foster understanding and promote the co-participation of professionals and health users.

The complexity of PHC also requires new professional profiles to meet community needs. The growing challenge of educational institutions is to prepare professionals to work at different levels of the health system, especially in PHC, in line with the system, teamwork, comprehensive health care, horizontal and more focused on the work process^(^ 18 ^)^.

Professionals, including health service managers, reported the need for more staff training. Professionals reported that they do not come prepared to work in the Family Health Strategy nor to accomplish humanizing practices. According to the professionals, this unpreparedness was related with the lack of such contents in undergraduate and continuing education. To work with extended concepts of health and care in PHC, the concepts of health and care widespread trough graduation and training courses need to be reconsidered. 

It is worth noting the Flexnerian influence in the formation of health professionals, based on the mechanical, biological and technical aspect, and complain-conduct practice^(^ 24 ^)^. Teaching of humanizing practices also suffers from the influence of scientific biomedicine. The rationalist, mechanistic and dualistic model of scientific rationality, dominant in health, prevents the recognition of other "truths" about health care, contrary to the reductionism of clinical pathology and mechanical physiology^(^ 25 ^)^.

In relation to continuing education, a pedagogical intervention in two Health Centers with themes about health system and humanizing practices showed that, after one year, there was an impact on management and work processes of some practices, with improved access and better relationship between professionals and community^(^ 26 ^)^. 

A health team needs technical, caring and management competence; demands beyond basic training, other skills that involve extended listening, speaking and teamwork^(^ 26 ^)^. This is the case of the skills related to nursing for universal health coverage. Moreover, there is a demand for training and continuing education, linking theory and practice, in order to build nurses and midwives' qualifications to become effective leaders and managers, as proposed by the strategic directions for nursing and midwifery education, training and career development^(^ 27 ^)^.

Regarding the provision of humanizing practices, in some services, the entire health team or just some professionals were involved with user embracement in order to increase access, bonding and accountability towards the community. In other units, user embracement was not deployed in order to reorganize the work process, but professionals were seeking to improve the care process and the relationship with patients through integral and universal care and extended listening.

 Professionals reported problems in the provision of humanizing practices. Results indicated that, often, the outcome of user embracement when mistaken for Emergency Care was medical consultation or medication, thus moving away from the goal of humanizing practices, which is to increase professional-patient bonding and meet community needs by delivering people-centered service. User embracement demands dialogue and qualified listening to result in a positive work environment, empowerment and teamwork.

Professionals reported that the provision of humanizing practices, especially the implementation of user embracement, resulted in changes in teamwork. The results indicated that the nursing staff is often confronted with user embracement with the presence of nursing technicians and auxiliaries. The user embracement team also includes community health agents, social service professionals and dentist auxiliaries. Nurses participate in the user embracement team and often play a role as rear to nursing team and community health agents. Also at the rear are physicians and dentists, who deliver support and advice to the user embracement team, however without presenting major changes in their work practice.

Workers of a multidisciplinary team in general realize that health work is done by different professionals in installments, but often do not realize that the absolute autonomy of a professional in relation to other workers and users hinders the construction of shared work^(^ 28 ^)^.

This was the case reported by some professionals in relation to those physicians who had difficulty participating in the user embracement and isolated their activities in PHC. But it was different from what was reported in relation to some members of the nursing staff and other professionals who have managed to put on teamwork and expand their shares in the unit through user embracement.

Professionals reported that the implementation of user embracement brought important changes in the practice of non-medical professionals in PHC, especially for social workers and nursing professionals who broaden their actions regarding health care, thus meeting a central idea of user embracement, which is to take the doctor's role as the only protagonist of care and extend the clinic conducted by other professionals^(^ 29 ^)^.

The Primary Health Care setting demands mutual responsabilization and integrated care, as well as understanding that user embracement is not screening and that complaint-conduct should not be the goal of health care teams. Professionals who come into contact with the humanizing practices had enlarged conceptions of health and care. But this expansion has not always been enough to change the working logic in the units and achieve universal health coverage.

Universal health coverage is an aspirational concept. It establishes what is to be achieved but little about how to get there. The first step is building a health workforce that is both fit for the purpose and fit to practice^(^ 30 ^)^. 

Some of the Strategic Directions for Strengthening Nursing and Midwifery Services^(^ 4 ^)^ can help and achieve universal health coverage. These are: strategies developed to encourage individuals, families and communities to play a more proactive part in assessing health-care needs and the effectiveness of service provision; standards of practice for people-centered care incorporated into quality health service delivery; development and implementation of PHC models led by nurses and midwifes; tools and models to improve the quality of practice, especially within PHC; research-based changes in nursing and midwifery practices to improve health services and outcomes; interprofessional and multisectoral collaboration strengthened to maximize the contribution of nurses and midwifes to health and development goals^(^ 4 ^)^.

Results related to midwives were not identified in this systematic review. Midwifery practice is also discussed in the Brazilian national policy towards Humanization practices; this scope is known as Child-birth Humanization and it defends respect for women's rights (sexual and reproductive, to universal access, to available technology); respectful treatment from providers; pain relief and prevention of iatrogenic pain^(^ 31 ^)^. 

A randomized controlled trial^(^ 32 ^)^ that examined the impact of supplementary prenatal care delivered by nurses in a community-based population related this practice to positive health outcomes for pregnant women. Based on this study^(^ 32 ^)^, overall skills, training and education requirements for universal health coverage could be identified. The overall skills were: holistic and universal approach that acknowledges physical, emotional and spiritual elements of pregnancy; provide support and address issues related to nutrition, lifestyle, food safety, psychosocial health and abuse, potential medical complications and exercise, multilingual and culturally appropriate care. Training and education requirements were community health, training in prenatal care and post natal follow-up, total person approach, humanistic perspective on learning, comprehensive pregnancy care, solution-focused counselling approach, community as partner approach, abuse screening and referral.

**Figure 3:**
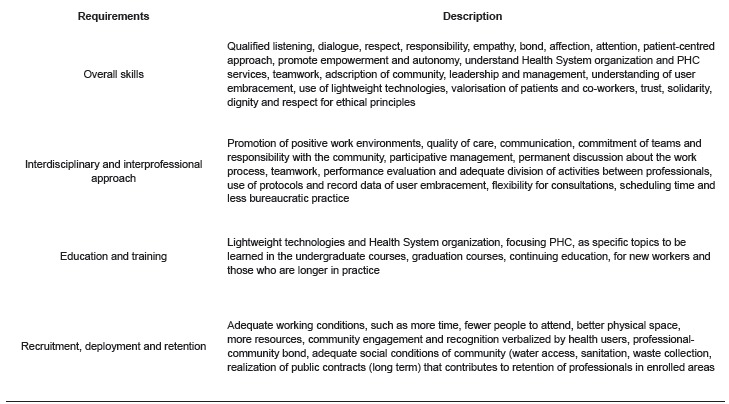
- Nursing requirements for universal health coverage

A broad range of nursing services were presented in the findings of this systematic review, such as nursing consultation, management of health units, orientation of nursing assistants, auxiliaries and community health agents, responsibilities related to vaccines, home visits, organization of therapeutic groups, participation in community health councils. In order to deliver these services and achieve universal health coverage, a list of requirements is presented in Figure 3.

Nursing challenges for universal health coverage, based on the requirements presented, are related to education and training, adequate working conditins in PHC and clear role clarification. These challenges are intrinsically related, since it is education and training together with working conditions that help to define nursing role in PHC.

Education and training considering expanded concepts of health and care, adequate working conditions in PHC, with teamwork and positive environment, are fundamental to recognize and reaffirm the role of nursing related to quality of life promotion and people-centered care. Nursing role in PHC is beyond treating patients with a disease, the demand of care might be for guidance, personal autonomy and empowerment, for patients to take care of themselves and their relatives. 

Among the studies included in the systematic review, a nurse in a health care unit that had no humanizing practices reported that there was nothing else to do for a patient with a terminal illness (A26). This is a demonstration of the nurse's mismatch with her professional role. Nursing goes beyond diagnosis, being a nurse is taking care of someone who has no capacity to do so at every stage of life and death process.

However, it is understood that the expanded concepts of health and care are contrary to the fragmented and medical-focused conception currently present in many workplaces, presented in the media and taught in several health courses. Therefore, it is necessary to invest in multidisciplinary teamwork, in deploying user embracement in the units, both for patients and professionals, privileging therapeutic areas and the use of soft technologies, to promote moments of encounter that create bonds and improve recognition of nursing by patients, community and other professionals.

Overall, the results from this systematic review are in accordance with some of the strategies towards Universal Health Coverage proposed by the 6^th^ Global Forum on Nursing and Midwifery. These are: policies that encapsulate the vision of UHC to ensure integrated people-centered services; educational approach towards quality and relevance of the nursing and midwifery workforce to meet the local and national changing health needs; interventions that lead to improved access to health care services and strategies that support collaborative partnerships to minimize barriers to health services^(^ 33 ^)^. 

One limitation of the study is that the majority of the studies included in the review are from Brazil. Nevertheless, results may be applied to different realities, especially to low and middle income countries and health systems facing critical health and social challenges, since these are the challenges of Brazilian primary health care. Humanizing practices can enhance the care promoted by nursing and its responsiveness towards the implementation of universal health coverage.

Nursing as a social practice demands reflection on the complexity of social issues and health, in line with the plurality of current society. Nurses must combine, in daily work, principles and values with competence, in an atmosphere of co-responsibility and care^(^ 34 ^)^.

## Conclusions

Nursing challenges for universal health coverage are related to education and training, adequate working conditions in PHC and clear role clarification in PHC. It is necessary to overcome difficulties, such as fragmented concepts of health and care, and invest in multidisciplinary work teams, community empowerment, professional-health user bond, user embracement, use of soft technologies, to promote quality of life, holistic care and improve the recognition of nursing by patients and other professionals.

The quality of the findings in this systematic review can be classified as low evidence, since most studies used interviews and focus groups as the primary method for collecting results. Nevertheless, based on the perceived benefits presented by the professionals in relation to humanizing practices into Primary Health Care, it is possible to make a strong recommendation towards this practice. Evidence suggests that nursing's frontline role in PHC for UHC can benefit from humanizing practices and from a more integrative conception of health and care. 
